# Standard coagulation tests are superior to thromboelastometry in predicting outcome of patients with liver cirrhosis

**DOI:** 10.1371/journal.pone.0236528

**Published:** 2020-07-28

**Authors:** Jassin Rashidi-Alavijeh, Ayse S. Ceylan, Heiner Wedemeyer, Martin Kleefisch, Katharina Willuweit, Christian M. Lange

**Affiliations:** Department of Gastroenterology and Hepatology, University Hospital Essen, University of Duisburg-Essen, Essen, Germany; University of Navarra School of Medicine and Center for Applied Medical Research (CIMA), SPAIN

## Abstract

**Background and aims:**

Thromboelastometry (TEM) is superior to standard coagulation tests in the management of bleedings / invasive procedures in patients with liver cirrhosis. In contrast, the role of TEM as a prognostic parameter in liver cirrhosis is not well established. We therefore aimed to assess the role of TEM in predicting survival of outpatients with liver cirrhosis.

**Methods:**

TEM was performed in consecutive outpatients with liver cirrhosis admitted in 2018 and 2019 to the University Hospital Essen. Associations with transplant-free survival were assessed in regression models.

**Results:**

A number of 145 outpatients with liver cirrhosis were included, of whom 27 received a liver transplant (N = 7) or died (N = 20) within 6 months of follow-up. None of the TEM values was associated with transplant-free survival in this cohort. However, as expected, the classical coagulation tests INR (OR = 8.69 (95% CI 1.63–46.48), *P* = 0.01), PTT (OR = 1.15 (95% CI 1.04–1.27), *P*<0.01), as well as antithrombin (OR = 0.96 (95% CI 0.94–0.99), *P*<0.01), and protein C (OR = 0.96 (95% CI 0.92–0.99), *P*<0.01) were significantly associated with transplant-free survival.

**Conclusion:**

In contrast to the superiority of TEM over classical coagulation tests to guide transfusion of blood products in patients with liver cirrhosis, TEM has no relevance in predicting mortality in outpatients with liver cirrhosis.

## Introduction

Liver cirrhosis is the final common path of many different chronic liver diseases, resulting in severe complications with high morbidity and mortality. In central Europe, liver cirrhosis is estimated the fourth most common cause of death. [1, 2] For patients with end-stage liver cirrhosis, liver transplantation (LT) is an important and often the only option for improving survival. [3] Due to insufficient numbers of donors, recipients of LT have to be chosen carefully. For this reason, prediction of mortality of patients with liver cirrhosis is of crucial importance.

There are different models for prediction of mortality of patients with cirrhosis, the CLIF consortium acute-on-chronic liver failure (ACLF) score (CLIF-C ACLFs), the CLIF consortium acute decompensation score (CLIF-C ADs), and the model of end-stage liver disease (MELD) being the most important of them. The CLIF-C ACLFs is used for patients with ACLF, which is characterized by acute decompensation of liver cirrhosis in combination with specific organ failures and high short-term mortality. [4, 5] The CLIF-C ADs, on the other hand, is predicting prognosis for patients with acute decompensation of liver cirrhosis without organ failure. [6] The MELD score is calculating estimated survival by utilization of three laboratory values (creatinine, bilirubin, INR) and is applied for prioritization of LT candidates in many countries. [7–9] Interestingly, both CLIF-C ACLFs and MELD are including the international normalized ratio (INR) as a marker for coagulopathy in their calculations, which highlights the importance of cirrhosis-associated coagulopathy in predicting prognosis of patients with liver cirrhosis.

The coagulopathy of advanced liver cirrhosis is characterized by decreased levels of numerous pro-coagulatory factors, resulting in deranged coagulation values in standard laboratory tests to assess coagulation, like INR or aPTT. [10, 11] Yet, there are important exceptions, namely von Willebrand-factor and factor VIII, which are increased in liver patients with advanced liver cirrhosis. [12] Furthermore, it is well known that synthesis of different anticoagulants such as protein C or antithrombin is significantly impaired as well. [13, 14] These alterations of pro- and anticoagulatory factors result in a still existing hemostatic balance despite deranged laboratory tests, [15] which can even lead to an increased risk of thromboembolic events. [16, 17] For these reasons, standard laboratory tests for coagulation have limited significance regarding characterization of the hemostatic situation of liver cirrhosis patients and the prognosis of potential bleeding events, which is contrasting the accuracy of these tests in predicting prognosis of patients with liver cirrhosis. [18, 19]

Thromboelastometry (TEM) is a dynamic bedside device which measures viscoelastic properties of whole blood specimens and is assumed to be a relevant alternative for assessing coagulation in the management of bleedings and before interventions in patients with liver cirrhosis, although it was primarily used for guiding transfusion in different surgical procedures, such as liver transplantation. [20–23] TEM can also be used to guide substitution of coagulation factors in patients with ACLF. [24] Tripodi *et al*. identified a moderate correlation between some TEM values (e.g. MCF) with the Child-Pugh-score in patients with stable liver cirrhosis. [25] Hypocoagulable features of TEM in liver cirrhosis patients were measured in different other studies, too, although some studies revealed normal TEM parameters in stable cirrhosis. [26–28] Blasi *et al*. recently demonstrated hypocoagulability in ACLF patients, which was associated with higher short-term mortality. [29]

Taken together, TEM (in contrast to INR) appears to be of high value in the management of bleedings and interventions in patients with liver cirrhosis, though the role of TEM in predicting outcomes of patients with liver cirrhosis is less clear. We therefore aimed to determine the relevance of TEM parameters in predicting survival of outpatients with liver cirrhosis.

## Patients and methods

### Patients

Between September 2018 and July 2019, consecutive outpatients with liver cirrhosis admitted to the University Hospital Essen were included in the present study. The diagnosis of liver cirrhosis was based on histopathology or a combination of clinical, laboratory and imaging findings (ultrasound and transient elastography or share wave elastography). Acute decompensation of liver cirrhosis and ACLF were diagnosed according to the ACLF-criteria proposed by the CLIF-EASL consortium [5]. Patients were excluded if they were younger than 18 years, in case of pregnancy or breastfeeding, presence of hepatocellular carcinoma (HCC) beyond Milan criteria, presence of infection with human immunodeficiency virus (HIV), or therapy with anticoagulants.

Routine laboratory testing as well as TEM was done at baseline of study inclusion. Demographic and clinical characteristics, including age, sex, body mass index (BMI), origin of liver cirrhosis and presence or absence of portal vein thrombosis, ascites, hepatic encephalopathy, diabetes and nicotine consumption were recorded. Patients were followed for at least 12 months. The study was conducted in accordance with the Helsinki Declaration of 1975 and was approved by the local ethics committee of the University Hospital Essen, Germany (ethics grant number: 15 6648 BO). In accordance with the local ethics committee, patient consent was not required.

### Thromboelastometry

Blood was taken by clean venepuncture upon presentation in our hepatology outpatient clinic. TEM was performed immediately with a rotational thromboelastometry (ROTEM) delta system (Tem Innovations, Munich, Germany) [30] in accordance to the instructions of the manufacturer. We included testing of EXTEM, INTEM and FIBTEM assays. EXTEM assay represents the extrinsic coagulation pathway and is comparable to prothrombin measurement. In the case of EXTEM assay, coagulation is induced by adding recombinant tissue factor to citrated whole blood. FIBTEM assay is performed as EXTEM assay with addition of cytochalasin D, a platelet inhibitor, thereby measuring fibrin polymerization. INTEM analysis represents the intrinsic coagulation system and is analogue to aPTT measurement. In this case, coagulation is induced by adding ellagic acid as a contact activator to citrated whole blood.

Parameters which were assessed for EXTEM and INTEM analysis were clotting time (CT, time from starting the assay to initiation of clot formation), clot formation time (CFT, time from clotting to reaching an amplitude of 20 mm), alpha angle (velocity of clot formation) and maximum clot firmness (MCF, maximum amplitude of the clot in the graphical trace in mm). Regarding FIBTEM, only MCF was assessed. For analysis of clot lysis, lysis was assessed at 30 (Ly30) and 60 (Ly60) minutes by measuring the percentage of clot reduction at these time points. Maximum lysis (ML) represents the percentage of decrease of amplitude between the maximum and minimum MCF.

### Statistical analysis

Nominal data were depicted as absolute numbers and percentages, metric variables were summarized as means and standard deviation. Associations between transplant-free survival, different laboratory values and values of TEM were assessed in logistic regression models. After univariate analyses, multivariate analyses were performed for significant associations. Multivariate analyses were obtained by using backward selection, using a *P* value < 0.15 for removal from the model. *P*-values < 0.05 were considered to be significant. Survival curves were estimated by the Kaplan-Meier method. Significance was calculated by cox´s regression model.

## Results

### Patient characteristics

A total of 145 outpatients with liver cirrhosis were included in this study. Mean patient age was 53 years (range, 20–69) and 62% of patients were male. The most frequent etiology of liver cirrhosis was alcoholic liver cirrhosis (40%), chronic hepatitis B or C (17%), followed by primary biliary cholangitis (PBC) / primary sclerosing cholangitis (PSC) (16%), and non-alcoholic steatohepatitis (NASH) (6%). Twenty-seven of these patients died (N = 20) or received LT (N = 7) during follow-up, while 118 survived without LT. The group of patients who died or received LT showed higher rates of portal vein thrombosis (15%), ascites (78%), and hepatic encephalopathy (52%) compared to those who survived without LT (8%, 55% and 23%, respectively). More detailed demographic and clinical characteristics are presented in Table 1.

**Table 1:** Baseline and demographic characteristics.

	Total (n = 145)	Survivors (n = 118)	Death/LT (n = 27)	P-Value
Male sex, n (%)	90 (62)	71 (60)	19 (70)	0.3
Age (years), mean (range)	53 (20–69)	52 (20–69)	55 (34–67)	0.4
BMI (kg/m^2^), mean (range)	26 (16–39)	26 (17–39)	27 (16–39)	0.9
**Origin of cirrhosis**				
Alcohol, n (%)	58 (40)	47 (40)	11 (41)	0.9
HCV/HBV, n (%)	24 (17)	17 (14)	7 (26)	0.1
PBC/PSC, n (%)	23 (16)	20 (17)	3 (11)	0.5
NASH, n (%)	8 (6)	4 (3)	4 (15)	0.02
AIH, n (%)	5 (3)	5 (4)	0 (0)	0.3
Other, n (%)	27 (19)	25 (21)	2 (7)	0.1
Portal vein thrombosis, n (%)	13 (10)	9 (8)	4 (15)	0.2
Ascites, n (%)	85 (59)	64 (55)	21 (78)	0.02
Hepatic encephalopathy, n (%)	41 (29)	27 (23)	14 (52)	0.003
Diabetes, n (%)	35 (24)	30 (26)	5 (19)	0.4
Nicotin consumption, n (%)	92 (65)	78 (68)	14 (52)	0.2
**Laboratory parameters (mean, SD)**				
Leukocytes (per nL)	5.9 (3.1)	5.6 (2.3)	7.2 (5.2)	0.06
Hemoglobin (g/dl)	11.9 (2.0)	12.1 (1.9)	11.2 (2.0)	0.07
Platelets (per nL)	138 (97.3)	141 (101)	123 (75)	0.3
Sodium (mmol/L)	138 (3.7)	139 (3.5)	137 (3.7)	0.006
Potassium (mmol/L)	4.2 (0.5)	4.2 (0.4)	4.2 (0.5)	0.7
Calcium (mmol/L)	2.2 (0.2)	2.2 (0.2)	2.2 (0.2)	0.3
Creatinine (mg/dL)	1.1 (0.6)	1.1 (0.6)	1.2 (0.5)	0.2
25-hydroxyvitamin D (ng/mL)	17 (11)	18 (10)	13 (6)	0.06
Bilirubin (mg/dL)	2.1 (1.9)	1.8 (1.5)	3.5 (2.5)	0.0001
AST (U/L)	61 (92)	56 (96)	81 (65)	0.0003
ALT (U/L)	53 (113)	54 (124)	50 (36)	0.1
γGT (U/L)	161 (219)	160 (213)	166 (247)	0.9
AP (U/L)	182 (146)	176 (140)	208 (167)	0.1
Albumin (g/dL)	3.7 (0.7)	3.8 (0.6)	3.2 (0.6)	0.0001
CRP (mg/dL)	1.3 (2.0)	1.2 (2.1)	1.7 (1.5)	0.006
INR	1.2 (0.2)	1.16 (0.2)	1.34 (0.2)	<0.0001
aPTT (seconds)	30.0 (4.2)	29.4 (3.4)	32.8 (6.0)	0.0009
Fibrinogen (mg/dL)	262.7 (93.6)	267.5 (84.4)	241.6 (124.1)	0.05
Antithrombin III (%)	74.2 (26.8)	78.5 (25.2)	55.0 (24.9)	<0.0001
Protein C (%)	64.9 (29.1)	69.5 (28.4)	44.2 (22.4)	<0.0001
Protein S (%)	82.0 (23.9)	83.5 (24.2)	75.6 (21.2)	0.1

AIH, autoimmune hepatitis; ALT, alanine aminotransferase; AP, alkaline phosphatase; aPTT, activated partial thromboplastin time; AST, aspartate aminotransferase; BMI, body mass index; CRP, C-reactive protein; γGT, gamma-glutamyltransferase; HBV, hepatitis B virus; HCV, hepatitis C virus; INR, international normalized ratio; LT, liver transplant; NASH, non-alcoholic steatohepatitis; PBC, primary biliary cholangitis; PSC, primary sclerosing cholangitis.

### Analysis of TEM values as predictors of transplant-free survival

Baseline TEM values of patients who survived or who died / were transplanted during follow up are shown in Table 2. Overall, mean TEM values were rather comparable to published TEM values in healthy individuals. [31] One can note that mean TEM values were almost similar between patients who survived or who died / were transplanted during follow-up. Furthermore, none of the TEM parameters showed a significant association with transplant-free survival in logistic regression analysis (Table 3).

**Table 2:** Thromboelastometry test results.

	Total (n = 145)	Survivors (n = 118)	Death/LT (n = 27)	P-value
**TE values (mean, SD)**				
**EXTEM**				
CT (seconds)	60 (13)	61 (14)	54 (7)	0.4
CFT (seconds)	105 (61)	105 (64)	104 (47)	0.6
MCF (mm)	58 (10)	58 (10)	56 (8)	0.6
Alpha Angle (°)	73 (8)	73 (9)	73 (7)	0.5
**INTEM**				
CT (seconds)	185 (21)	186 (23)	180 (15)	0.07
CFT (seconds)	99 (59)	99 (62)	100 (49)	0.9
MCF (mm)	55 (10)	55 (10)	53 (8)	0.4
Alpha Angle (°)	73 (7)	73 (7)	73 (6)	0.9
**FIBTEM**				
MCF (mm)	17 (8)	18 (9)	15 (5)	0.1
**Clot Lysis**				
Ly30 (%)	100 (1)	100 (1)	99 (1)	0.7
Ly60 (%)	91 (4)	91 (4)	89 (4)	0.9
ML (%)	12 (5)	12 (5)	13 (4)	0.7

CFT, clot formation time; CT, clotting time; MCF, maximum clot firmness; ML, maximum lysis.

**Table 3:** Univariate analysis of association between TE parameters and mortality/transplantation in patients with liver cirrhosis.

	Univariate analysis	
	OR (95% CI)	P
**EXTEM**		
CT	0.99 (0.96–1.02)	0.68
CFT	1.00 (0.99–1.00)	0.73
MCF	0.99 (0.95–1.03)	0.65
Alpha Angle	0.98 (0.94–1.04)	0.54
**INTEM**		
CT	1.00 (0.97–1.03)	0.77
CFT	1.01 (1.00–1.03)	0.11
MCF	1.00 (1.00–1.01)	0.47
Alpha Angle	1.00 (0.93–1.05)	0.63
**FIBTEM**		
MCF	1.09 (0.99–1.19)	0.08
**Clot lysis**		
Ly30	1.11 (0.63–1.97)	0.71
Ly60	1.00 (0.89–1.12)	1.00
ML	1.04 (0.94–1.14)	0.48

CFT, clot formation time; CT, clotting time; MCF, maximum clot firmness; ML, maximum lysis

### Analysis of standard coagulation parameters as predictors of transplant-free survival

**Fig 1:**
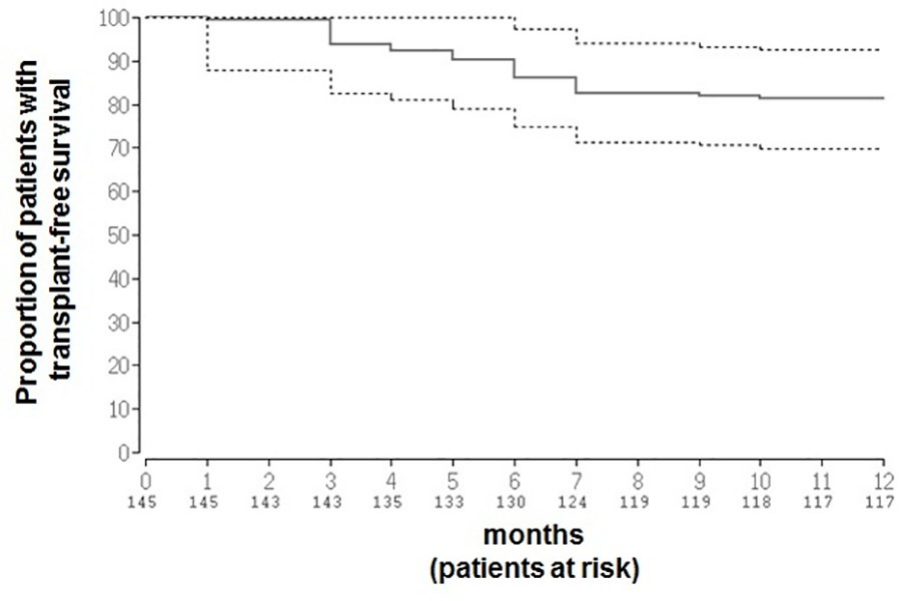
Kaplan Meier curve of transplant-free survival. Proportions (solid line) and confidence intervals (dotted lines) of patients surviving without liver transplantation during 12 months of follow-up are shown. Bilirubin (beta = 0.17, 95% CI = -0.01–0.36, P = 0.06) and protein C (beta = -0.03, 95% CI = -0.05 - -0.005, P = 0.01) were independently associated with transplant-free survival in Cox regression analysis.

We analyzed different laboratory parameters and in particular different standard coagulation parameters for predicting transplant free survival. In univariate analysis, INR (P = 0.01, OR = 8.69, 95% CI = 1.63–46.48), aPTT (P<0.01, OR = 1.15, 95% CI = 1.04–1.27), Antithrombin III (P<0.01, OR = 0.96, 95% CI = 0.94–0.99), protein C (P<0.01, OR = 0.96, 95% CI = 0.92–0.99) and bilirubin (P<0.01, OR = 1.48, 95% CI = 1.48–1.84) showed significant association with transplant-free survival. The association between protein C (P = 0.01, OR = 1.00, 95% CI = 0.94–0.99) and bilirubin (P = 0.05, OR = 1.27, 95% CI = 1.00–1.62) with transplant-free survival remained significant after multivariate analysis (Table 4). These variables were also associated with transplant-free survival in Cox-regression analysis (Fig 1).

**Table 4:** Uni- and multivariate analyses of association between different variables and mortality/transplantation in patients with liver cirrhosis.

	Univariate analysis		Multivariate analysis	
	OR (95% CI)	P	OR (95% CI)	P
Age (years, continuous)	1.02 (0.99–1.08)	0.19		
Male sex, presence	0.70 (0.28–1.76)	0.44		
**Laboratory values (continuous)**				
Leukocytes (per nL)	1.12 (0.94–1.34)	0.21		
Hemoglobin (g/dl)	0.85 (0.68–1.07)	0.17		
Thrombocytes (per nL)	1.00 (0.99–1.00)	0.51		
INR	8.69 (1.63–46.48)	**0.01**		
aPTT (seconds)	1.15 (1.04–1.27)	**<0.01**		
Fibrinogen (mg/dL)	1.00 (0.99–1.00)	0.33		
Antithrombin III (%)	0.96 (0.94–0.99)	**<0.01**		
Protein C (%)	0.96 (0.92–0.99)	**<0.01**	0.99 (0.94–0.99)	**0.01**
Protein S (%)	0.99 (0.97–1.01)	0.20		
Creatinine (mg/dL)	1.23 (0.69–2.22)	0.48		
Bilirubin (mg/dL)	1.48 (1.19–1.84)	**<0.01**	1.27 (1.00–1.62)	**0.05**
ALT (U/L)	1.01 (1.00–1.02)	0.27		
γGT (U/L)	1.00 (1.00–1.00)	0.83		
CRP (mg/dL)	1.09 (0.91–1.31)	0.34		

ALT, alanine aminotransferase; aPTT, activated partial thromboplastin time; CRP, C-reactive protein; γGT, gamma-glutamyltransferase; INR, international normalized ratio

## Discussion

The main finding of the present study is that TEM parameters are not predictive for transplant-free survival of outpatients with liver cirrhosis. This is in contrast to the importance of classical coagulation tests, namely INR, to predict mortality of patients with liver cirrhosis, and to the relevance of TEM in the management of bleedings and periprocedural bleeding risk in these patients.

Coagulopathy of liver cirrhosis–one of the hallmarks of the disease—is characterized by reduced plasma levels of most coagulation factors and important natural anticoagulants such as protein C and antithrombin, but also by a substantial increase of procoagulants factor VIII and von-Willebrand factor. [10] Consequently, patients with advanced liver cirrhosis are at risk for both bleeding and thromboembolic events, which can–however–be poorly predicted by standard coagulation tests. [10] Therefore, it was plausible to assess the value of assays such as TEM, which directly measure blood-clotting capacity in order to improve the management of bleedings and invasive procedures. In a randomized controlled study of patients with liver cirrhosis and severe coagulopathy (defined as INR >1.8 and platelets < 50/ nl), usage of TEM in comparison to standard coagulation tests significantly reduced the rate of transfusion of blood products before invasive procedures (16.7% versus 100% transfusion rate, P<0.0001), without increasing the (generally very low) risk of bleedings. [26] The superiority of TEM to guide the periprocedural need of transfusions of blood products in patients with liver cirrhosis was confirmed in other studies [32], including an analysis of patients with ACLF. [24] In addition, TEM has been shown to be superior in the management of variceal and non-variceal bleedings in patients with liver cirrhosis. In these scenarios, usage of TEM resulted in a decreased amount of transfused blood products without affecting bleeding control rate or survival. [33, 34] In line with these findings, a recent study has shown that TEM and INR did not correlate well in patients with liver cirrhosis and claimed the TEM may better reflect hemostatic abnormalities and bleeding risk in these patients. [35]

The superiority of TEM over standard coagulation tests to manage cirrhosis-associated coagulopathy in the setting of invasive procedures and bleedings may indicate a value of TEM in predicting outcome of patients with liver cirrhosis. Yet, virtually no association between TEM values and transplant-free survival has been observed in our study of outpatients with liver cirrhosis, whereas classical coagulation tests, namely INR and PTT, but also protein C and antithrombin, were significantly associated with this endpoint. Our data are in line with a previous cross-sectional study evaluating TEM at the time of evaluation for liver transplantation, which did not show any correlation between TEM values and Child-Pugh- or MELD-score [36], as well as with another analysis of outpatients with liver cirrhosis in whom no association between survival and TEM was observed. [37]

At first glance, the fact that TEM is superior in management of bleeding events but fails completely in prediction of survival in outpatient cohorts seems surprising. The reason why parameters like INR and protein C are superior in prediction of survival might be that classical coagulation parameters reflect well the degree of liver synthesis failure, although their value in predicting bleeding events seems to be negligible due to a rebalanced hemostatic process, [15] In this rebalanced condition, hemostasis is still inconspicuous, leading to normal TEM values.

In contrast, in the situation of acute decompensation of liver cirrhosis and ACLF, associations between survival and TEM values have been observed. Hypocoagulable features in the situation of acute decompensation and ACLF, i. e. a delayed clot formation time (CFT_EXT_) and decreased clot firmness (MCF_EXT_), are associated with higher mortality. [29]

Importantly, in patients with acute decompensation or ACLF, hypocoagulable TEM values appear to be affected by the degree of systemic inflammation. [29] For this reason, it is not surprising that CFT_EXT_ and MCF_EXT_ are not deranged in our outpatient cohort with compensated cirrhosis, but are altered in studies with patients with decompensated cirrhosis or ACLF.

In conclusion, it appears plausible that TEM is not suitable to predict outcomes of relatively stable patients with cirrhosis, in whom classical coagulation parameters reflect well the degree of liver synthesis failure, but may become important in patients with ACLF in whom abnormal TEM values partially reflect the magnitude of inflammation or infections, i.e. the drivers of organ failures in these patients.

## Abbreviations

LT: Liver transplantation
ACLF: Acute-on-chronic liver failure
CLIF-C ACLFs: CLIF consortium ACLF score
CLIF-C Ads: CLIF consortium acute decompensation score
MELD: Model of end-stage liver disease
INR: International normalized ratio
TEM: Thromboelastometry
HCC: Hepatocellular carcinoma
HIV: Human immunodeficiency virus
ROTEM: Rotational thromboelastometry
CT: Clotting time
CFT: Clot formation time
MCF: Maximum clot firmness
ML: Maximum lysis
PBC: Primary biliary cholangitis
PSC: Primary sclerosing cholangitis
